# Evaluating and implementing The CONNECT Program—A group-based telehealth intervention to reduce social isolation, loneliness, and mental health symptoms in adults 55**+** vs routine community programming: Study protocol for a randomized controlled trial

**DOI:** 10.1371/journal.pone.0336031

**Published:** 2025-11-11

**Authors:** Kira Kudar, Georgia Gopinath, Alex Ross, Robert Balshaw, Edwin Chau, Inga Christianson, Lesley Koven, Corey S. Mackenzie, Stacey Miller, Nancy Newall, Lyne Ouellet, Kathryn Sibley, Alina Sanina, Ronda Wedhorn, Kristin A. Reynolds

**Affiliations:** 1 Department of Psychology, University of Manitoba, Winnipeg, Manitoba, Canada; 2 Department of Community Health Sciences, University of Manitoba, Winnipeg, Manitoba, Canada; 3 Brella Community Services Society, Surrey, British Columbia, Canada; 4 Department of Clinical Health Psychology, University of Manitoba, Winnipeg, Manitoba, Canada; 5 A & O: Support Services for Older Adults, Winnipeg, Manitoba, Canada; 6 Department of Psychology, Brandon University, Winnipeg, Manitoba, Canada; 7 Department of Gerontology, St. Thomas University, Fredericton, New Brunswick, Canada; 8 Seniors Centre Without Walls Saskatchewan, Moose Jaw, Saskatchewan, Canada; Public Library of Science, UNITED STATES OF AMERICA

## Abstract

**Background:**

The population of adults aged 55 years and older in Canada is growing steadily to be one of the largest demographic groups, creating an aging society. Adults 55 + are facing dynamic changes in employment and finances, relationships, living situation, and health, which may contribute to increased psychosocial and psychological challenges, including social isolation, loneliness, depression, and anxiety. Importantly, when adults 55 + need mental health support, they are less likely to access this support compared to other demographics. When supports are grounded in the community and are available through telephone and video-conferencing, access can be improved. Developed in partnership with community organizations, The CONNECT Program is designed to improve the psychosocial well-being of older adults, focusing on psychological flexibility, and in this randomized controlled trial will be compared against routine community-based programming across Canada.

**Methods:**

This randomized crossover trial is embedded within a type 1 hybrid implementation-effectiveness design across four Canadian provinces. Adults aged 55+ with self-reported experiences of loneliness, social isolation, and/or mental health challenges are randomly assigned to begin with either the intervention (The CONNECT Program) or community-based programming, then cross over to the other condition. The CONNECT Program is a six-week, group-based telehealth intervention grounded in Acceptance and Commitment Therapy, self-compassion, theories of successful aging delivered via telephone or videoconference. The primary outcome is psychological flexibility; secondary outcomes include loneliness, social isolation, emotional support, anxiety, depression, and mental health literacy. Implementation outcomes will be assessed using the Proctor Framework.

**Discussion:**

The CONNECT Program is a novel community-based mental health intervention addressing the needs of adults 55 + who experience isolation, loneliness and mental health challenges. The results of this trial will strengthen support for a scalable mental health support for Canadians 55+ in a time of high need to promote health in later life.

**Trial registration**

The trial was registered on ClinicalTrials.gov: NCT07107906, 08/05/2025, https://clinicaltrials.gov/study/NCT07107906.

## Introduction

The aging of the Canadian population is particularly stark, with a 3.4% rise in the 65 + population from 2023 to 2024, due to the large baby boomer groups [1]. Older adults currently represent 18.9% of the Canadian population and expected to be between 21.9% and 32.3% of the Canadian population by 2073 [2]. This demographic trend emphasizes the critical importance of addressing related psychosocial problems, particularly the increasing threat of social isolation and loneliness, both of which are now recognized as significant public health problems. Social isolation refers to the “objective measure of social interactions and relationships [3], whereas loneliness refers to “subjective measurement of perceived social isolation or outcast” [3]. Recent data indicate that loneliness affects approximately one in six people globally, with a prevalence of 11.8% among adults aged 55+ [4]. A recent meta-analysis of 126 studies including 1,250,322 older adults found an overall loneliness prevalence of 27.6%. The highest rates were observed in North America (30.5%), among older women (30.9%) [5].

Such social disconnection has serious implications: it is linked to worse physical health, elevated risk of depression, accelerated cognitive decline, and increased mortality [4,6,7]. Though individuals may experience mental health problems such as anxiety and mood disorders in later life for a variety of reasons, social isolation and loneliness are associated with the development and persistence of such mental health challenges [8,9]. Loneliness is closely linked to depression in older adults, longitudinal data from the English Longitudinal Study of Ageing (ELSA) showed that increased loneliness predicted higher depressive symptoms over time [10]. Further, reduced experiences of belonging and meaningful connection contribute to both loneliness and depression [10] and loneliness has a moderate but significant effect on depression [5,11]. Meta-analytic results from Das and colleagues [12] showed that the older adults who did not socially participate were 2.07 times more likely of becoming depressed than those participated in social events, although, other literature reports no association of isolation and depression [13]. One possible pathway between social disconnectedness and mental health challenges was identified using a large sample of adults ages 57–85. Social isolation was found to predict loneliness, and this predicted increased depressive and anxiety symptoms. The reverse pathways were also statistically supported [14]. Analysis of pooled data from the 2015–2022 Canadian Community Health Survey (n = 151,755 adults aged 65+) found that, on average, 6.0% of older Canadians reported an anxiety disorder diagnosis [15]. A recent meta-analysis [16] estimated that 16.5% of older adults globally experience anxiety.

Community participation is linked to improved mental health [17–20] and lower rates of depression, anxiety, and cognitive impairment [21]. Using Canadian Longitudinal Study on Aging (CLSA) data (N = 51,338), our group found that frequent participation in community programs predicted better social support, cognition, life satisfaction, and physical and mental health outcomes [22]. Smale et al. (2022) [23] suggested that participation in and perceived access to leisure, recreational and cultural opportunities lower the risk of loneliness in older adults. Sense of belonging to the community, and especially overall sense of community, are the strongest mitigating factors against loneliness among older adults.

Community-based group programs have been employed for decades as a means of preventing social isolation among adults aged 55 + , involving activities like recreational opportunities, access to drop-in services and regular check-in calls. However, this might not fully address the needs of those 55 years or older who have mental health conditions or cannot come for in-person participation [24]. Community-based group interventions (e.g., Circle of Friends [25,26] the telephone-based Senior Center Without Walls [27], and “Neighbourhoods in solidarity” program in Switzerland [28] show varying levels of success reducing loneliness or increasing social connectedness. Nevertheless, substantial barriers persist for older adults with unmet mental health needs.

Older adults experience high rates of mental health issues, loneliness, and isolation, yet are less likely to seek professional help than younger groups, with about 70% of those with anxiety or mood disorders not accessing services [29,30]. In a survey of ~250 older Manitobans, 57% reported being unfamiliar with mental health treatments, underscoring the need for programs that improve mental health literacy and promote community-based support [17,31]. This may be indicative of adults 55 + preferring community-based services over the formal standard clinical care, which is why it is important to offer interventions in more familiar and accessible ways.

Telehealth innovations may serve as a valuable tool to support socially isolated older adults; a need that became particularly evident during the COVID-19 pandemic [24]. Research has shown that digital group interventions can effectively reduce loneliness and depression, as shown in a Zoom-based pilot RCT [32] and a 4-week program of empathy-focused phone calls that significantly reduced loneliness, depression, and anxiety among older adults [33]. These findings support a growing consensus on the mental health benefits of technology-enabled interventions for adults aged 55+ [34].

To integrate the strengths of community-based approaches with the accessibility of telehealth, we developed The CONNECT Program: a novel group-based intervention designed specifically to reduce social isolation, loneliness, and co-occurring mental health symptoms of depression and anxiety in adults aged 55 and over. The intervention is grounded in Acceptance and Commitment Therapy (ACT), which targets psychological flexibility as its core change process [35]. Also, The CONNECT Program integrates self-compassion approach [36], and psychosocial theories of successful aging [37,38].

The CONNECT Program was created through an academic-community partnership, initially in collaboration with A & O: Support Services for Older Adults (a community organization in Manitoba, Canada). We conducted a pilot study to evaluate the feasibility, acceptability, and preliminary effects of The CONNECT Program [39]. In a single-group pre/post pilot with older adult participants (recruited through A & O: Support Services for Older Adults, Winnipeg, Manitoba), The CONNECT Program was found to be highly feasible and acceptable. Qualitative feedback from post-program interviews was positive: participants highlighted the accessibility of The CONNECT Program – they appreciated the comfort and safety of joining sessions from home and noted that telephone delivery reduced typical age-related barriers (no travel needed, no exposure of disabilities) in accessing a support group. They also emphasized feelings of connectedness fostered by the program – reporting meaningful connections developed with the group facilitators and fellow group members, as well as the value of learning new information from the sessions. The quantitative pre- to post-program changes were promising, participants showed significant improvements in depression (d = 2.4), emotional support (d = 5.2), mental health literacy (d = 3.2), and psychological flexibility (d = 7.0), indicating large treatment effects [39,40].

To further understand the efficacy of The CONNECT Program within community settings, we are conducting a randomized control trial (RCT) and offering the intervention in Manitoba and other senior-serving organizations across Canada, including Brella Community Services Society in British Columbia, Senior Citizens Assistance Program in Saskatchewan, and department of Gerontology at the St. Thomson University in New Brunswick. To establish a comparison, we chose community-based service-as-usual programming as the comparator condition in this RCT. Participants randomized to the control group will receive the standard community programming offered by the partner organizations (service-as-usual), which may include social, educational, or recreational group sessions that are already offered by local organizations in person, by phone, or virtually. This comparator was selected because it represents a widely available, established model of psychosocial support for older adults across Canada. By using service-as-usual as the control, we can evaluate The CONNECT Program’s added value over and above the supports that older adults are already getting in their communities. Importantly, this design also ensures we are comparing the new program to a realistic alternative rather than to no intervention. Using service-as-usual also allows us to assess implementation under typical conditions – for example, in some regions the usual programs are phone-based, in others they might be virtual video sessions, or in-person, reflecting the range of formats currently in use.

Building on the pilot, the current study is a RCT to rigorously evaluate the CONNECT Program’s effectiveness and implementation. We have two objectives:

**Aim 1:** Examine the effectiveness of The CONNECT Program in improving key social and mental health outcomes in older adults.

**Aim 2:** Evaluate the community-based implementation of The CONNECT Program in multiple regions.

## Materials and methods

### Patient and public involvement, trial design

Before launching the trial, a few pre-implementation studies were conducted together with the partner community organisations across three provinces (British Columbia, Manitoba, Saskatchewan) and adult participants aged 55 + . We completed one virtual focus group per province with staff from each of the partner sites to gather input on how to adapt The CONNECT Program for different provinces [41–43]. This included feedback on revising the materials to reflect local needs, choosing the best delivery format (phone or videoconferencing), and designing a training and supervision process for community-based facilitators. We also held virtual focus groups and interviews with adults aged 55+ to learn about their mental health support needs, views on The CONNECT Program and implementation preferences [44].

This study uses a randomized crossover trial design, embedded within a type 1 hybrid implementation-effectiveness framework [45]. Participants are randomly assigned in a 1:1 ratio to begin either with The CONNECT Program or with the community-based programming. After completing the first condition, they will cross over to the other condition. Two sites will begin with videoconference mode and then switch to telephone mode, while the other two will follow the opposite sequence. In this RCT the effectiveness and implementation outcomes follow a superiority framework. Recruitment is ongoing in four Canadian provinces, with site-specific recruitment periods. The recruitment started on 8 September 2025, and is ongoing, with recruitment date for both primary and secondary end point collection expected to be collected by 30 September 2026.

### Participants, interventions, and outcomes

The trial will be conducted in community-based settings in four Canadian provinces (British Columbia, Manitoba, New Brunswick, Saskatchewan). Inclusion criteria for participants are: adults aged 55 years or older, able to speak, read, and understand English, can manage hearing or vision challenges well enough to participate in group conversations, self-reported experiences of loneliness, social isolation, and/or mental health challenges (including anxiety/depression symptoms). The exclusion criterion is: cannot communicate in English (verbal or written). Eligible facilitators include mental health professionals (e.g., social workers, psychologists, or geriatric clinicians) who are staff or volunteers of the partner organisations, as well as trained non-clinical staff or volunteers.

The CONNECT Program is a six-week, group-based telehealth intervention designed to reduce loneliness, social isolation, and co-occurring mental health symptoms (anxiety and depression) among adults aged 55 and older. Grounded in principles of ACT [35], self-compassion [36], and theories of successful aging [37,38], the program supports participants in building psychological flexibility, strengthening meaningful social connections, and engaging in values-based action. Each group meets once a week (6 weeks) for 90 minutes, either by telephone or via videoconferencing. Groups are typically 6–8 participants. Sessions follow a consistent structure, beginning with a grounding or mindfulness practice, followed by the introduction of core concepts drawn from ACT or self-compassion, group reflection, and discussion of real-life applications. Homework exercises are assigned each week, encouraging participants to practice new skills in their daily lives. These activities are supported by a structured participant workbook, which is available in both print and digital formats, depending on individual preference. The intervention was not tailored; all participants received the same materials and delivery format. The comparator condition consists of community-based programming that is already offered by participating partner organizations as part of their routine services. These programs may include group activities with a social, educational, or recreational focus, delivered by phone, videoconference, or in person. Programming varies slightly by site but reflects the standard community-based programs available to adults 55+ in each region.

**Fig 1:**
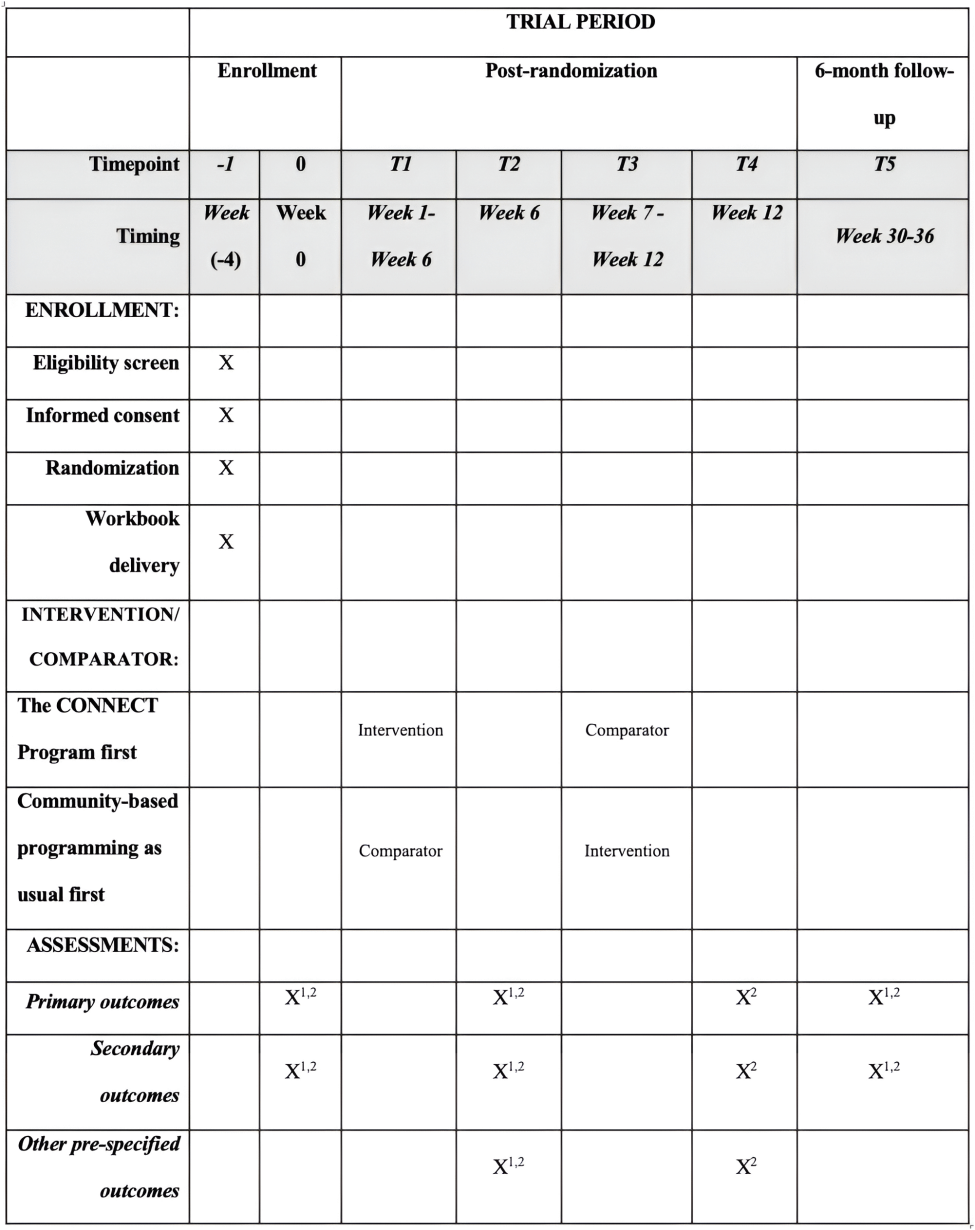
Schedule of enrollment, interventions, and assessments. X¹ - Participants who received The CONNECT Program first. X² - Participants who received The CONNECT Program second (after waitlist). Each participant is randomized to one of two treatment sequences – either starting with The CONNECT Program followed by community-based programming, or vice versa. A crossover occurs at the transition between week 6 and week 7. The 6-month follow-up will be conducted 24 weeks after each participant completes The CONNECT Program, which may fall between weeks 30 and 36 depending on their assigned treatment sequence.

Schedule of enrollment, interventions, and assessments is presented in Fig 1, following SPIRIT guidelines [46].

Prior to delivering the program, all facilitators complete a structured training program led by the research team. Training includes an introduction to the theoretical foundations of the program, facilitation skills for telehealth delivery, safety and crisis response planning, and an in-depth review of The CONNECT Program content and structure. Facilitators may also seek clinical supervision or consultation through weekly national meetings with the study team and other facilitators across sites during program delivery to support implementation challenges. All facilitators will take part in the same training and receive supervision support throughout the program to make sure its delivery is consistent and high-quality across locations.

Participation in the trial is voluntary; participants can withdraw at any time with no reason. If a participant experiences significant emotional distress or expresses suicidal thoughts, the facilitator will follow a safety protocol, which may include pausing the participant’s involvement and referring them to appropriate mental health support. Facilitators track weekly attendance and maintain regular contact with participants. Homework completion is encouraged and followed up on during sessions. Participants may continue any existing treatment, including medication or therapy to simulate real-world conditions, however, they are asked to avoid joining new mental health support groups during the trial to reduce interference with outcome interpretation, though adherence to this cannot be enforced. The effectiveness of The CONNECT Program will be examined through a set of primary and secondary outcomes, described below; implementation of the program will be assessed through other pre-specified outcome.

### Primary outcome

The CONNECT Program target, psychological flexibility, is the primary outcome and will be measured with the Acceptance and Action Questionnaire-II [47] (7-item self-report). Scores range from 7 to 49, with lower scores indicating greater psychological flexibility. Psychological flexibility is a core target of ACT and has been found to function as a transdiagnostic treatment process of change in psychotherapy [48]. Psychological flexibility is selected as the primary outcome in this trial because evidence shows that psychological inflexibility is strongly correlated with anxiety (r = 0.66, p < .001) and depression (r = 0.70, p < .001) in older adults [49], and facets of flexibility such as openness and awareness demonstrate medium-to-large associations with these symptoms [50]. Moreover, interventions enhancing flexibility, including mindfulness-based CBT (g = 0.55 for depression; g = 0.58 for anxiety) [51] and ACT, have been shown to reduce psychopathology and improve quality of life in older populations [52].

### Secondary outcomes

Secondary outcomes include changes from baseline in the following domains: loneliness**,** social isolation, emotional support, anxiety symptoms, depressive symptoms, and mental health literacy. All outcomes will be measured using validated self-report instruments.

### Loneliness

Loneliness will be measured with the De Jong Gierveld Loneliness Scale, a validated 6-item scale that assesses different aspects of loneliness (emotional and social) [53]. The total score ranges from 0 to 6, with higher scores indicating more loneliness. Loneliness heightens the risk of developing late-life depression and anxiety and is associated with numerous deleterious consequences for physical as well as cognitive health [54].

### Social isolation

Social isolation will be assessed using the PROMIS Social Isolation 8a short form [55], an 8-item standardized measure of perceived disconnection from others. Scores range from 8 to 40, with higher scores indicating greater social isolation. Social isolation is an objective risk factor for poor mental and physical health and is closely linked to late-life depression, anxiety, and cognitive decline [9,54].

### Emotional support

Emotional support will be assessed using the PROMIS Emotional Support measure [55], which evaluates the perceived availability of caring and understanding individuals in one’s life. Scores range from 4 to 20, with higher scores indicating greater emotional support. Greater emotional support is associated with reduced risk of depression, better coping with stress, and improved quality of life in older adults [21,22].

### Anxiety symptoms

The PROMIS Anxiety Short Form 4a [56], a validated 4-item self-report scale measure covering essential anxiety dimensions such as fear, worry, and nervousness, will be used to assess anxiety symptoms. Scores range from 4 to 20, with higher scores indicating greater severity of anxiety symptoms. Anxiety is prevalent among older adults and is strongly linked to social isolation and reduced quality of life [57,58].

### Depressive symptoms

Depressive symptoms will be measured using the PROMIS Depression Short Form 4a [56], a 4-item self-report instrument designed to assess some key features of depression (including sadness and hopelessness). Scores range from 4 to 20, with higher scores indicating greater severity of depressive symptoms. Subsyndromal depressive symptoms are prevalent in late life and are associated with reduced quality of life and increased health risks [59,60].

### Mental Health literacy

Mental health literacy will be assessed using the Brief Mental Health Literacy Scale [61], exploring knowledge and beliefs about mental disorders, help-seeking, and available treatments. Respondents rate their knowledge on a 5-point Likert scale (1 = not at all, to 5 = extremely). Total scores range from 4 to 20, with higher scores indicating greater perceived mental health literacy. Older adults typically have lower mental health literacy rates, compared with younger groups, which act as barriers to treatment access and self-identification of mental health problems [29,62].

### Other pre-specified outcomes

This trial also includes an examination of implementation outcomes, consistent with a type 1 hybrid effectiveness-implementation design. The implementation outcomes will be measured using the Proctor Framework [63] which defines eight components as follows: acceptability, adoption, appropriateness, cost, feasibility, fidelity, penetration, and sustainability.

### Acceptability of The CONNECT program

Acceptability is defined as the perception among implementation stakeholders that a given treatment, service, practice, or innovation is agreeable, palatable, or satisfactory [63]. Data will be collected through a post-program survey that includes Likert-scale and open-ended questions (with items developed based on [63]), an exit interview, the Intervention Content evaluation (adapted from [64]), and the Intervention Delivery Type evaluation (also adapted from [64]), and the Group Session Rating Scale [65], administered weekly during The CONNECT Program. In addition, a weekly facilitator check-in questionnaire will track dropout, attendance, and participant-reported reasons for not attending or discontinuing.

### Adoption of The CONNECT program

Adoption is defined as the intention, initial decision, or action to try or employ an innovation or evidence-based practice [63]. Data will be collected through a post-program survey that includes Likert-scale and open-ended questions (with items developed based on [63]. In addition, a weekly facilitator check-in questionnaire will track dropout, attendance, and participant-reported reasons for not attending or discontinuing.

### Appropriateness of The CONNECT program

Appropriateness is the perceived fit, relevance, or compatibility of the innovation or evidence-based practice for a given setting, provider, or consumer, and/or the perceived fit of the innovation to address a particular issue or problem [63]. Data will be collected through a post-program survey that includes Likert-scale and open-ended questions (with items developed based on [63], an exit interview, the Intervention Content evaluation (adapted from [64]), and the Intervention Delivery Type evaluation (also adapted from [64]). In addition, a weekly facilitator check-in questionnaire will track dropout, attendance, and participant-reported reasons for not attending or discontinuing.

### Feasibility of The CONNECT program

Feasibility is defined as the extent to which an innovation can be successfully used or carried out within a given agency or setting [63]. Data will be collected through a post-program survey that includes Likert-scale and open-ended questions (with items developed based on [63], an exit interview, the Intervention Content evaluation (adapted from [64]), and the Intervention Delivery Type evaluation (also adapted from [64]. In addition, a weekly facilitator check-in questionnaire will track dropout, attendance, and participant-reported reasons for not attending or discontinuing.

### Implementation Costs of The CONNECT program

Implementation costs are defined as the cost impact of an implementation effort [63]. Data will be collected through a post-program survey that includes Likert-scale and open-ended questions (with items developed based on [63]).

### Penetration of The CONNECT program

Penetration refers to the integration of an innovation within a service setting and its reach among intended users. At the recipient level, it is defined as the number of eligible individuals who use a service, divided by the total eligible population. At the setting level, it is the number of providers delivering the service, divided by the total number trained or expected to deliver it [63]. Data will be collected through a post-program survey that includes Likert-scale and open-ended questions (with items developed based on [63]).

### Sustainability of The CONNECT program

Sustainability is defined as the extent to which a newly implemented treatment is maintained or institutionalized within a service setting’s ongoing, stable operations [63]. Data will be collected through a post-program survey that includes Likert-scale and open-ended questions (with items developed based on [63]). In addition, a weekly facilitator check-in questionnaire will track dropout, attendance, and participant-reported reasons for not attending or discontinuing.

A detailed overview of the assessment schedule by timepoint and study week is provided in Table 1.

**Table 1:** Schedule for assessment administration.

	TRIAL PERIOD						
	Enrollment		Post-randomization				6-month follow-up
Timepoint	*−1*	0	*T1*	*T2*	*T3*	*T4*	*T5*
Timing	*Week *(−4)	Week 0	*Week 1 -Week 6*	*Week 6*	*Week 7 -Week 12*	*Week 12*	*Week 30–36*
Sociodemographics		X^1,2^					
Psychological flexibility (Acceptance and Action Scale; [47]) Acceptance and Commitment Scale		X^1,2^		X^1,2^		X^2^	X^1,2^
DeJong Gierveld Loneliness Scale [53]		X^1,2^		X^1,2^		X^2^	X^1,2^
Social Isolation (PROMIS Social Isolation 8a; [55]		X^1,2^		X^1,2^		X^2^	X^1,2^
Emotional Support (PROMIS Emotional Support; [55])		X^1,2^		X^1,2^		X^2^	X^1,2^
Anxiety (PROMIS Anxiety Short Form 4a; [56])		X^1,2^		X^1,2^		X^2^	X^1,2^
Depression (PROMIS Depression Short Form 4a; [56])		X^1,2^		X^1,2^		X^2^	X^1,2^
Mental Health Literacy (Brief Mental Health Literacy Scale; [61]		X^1,2^		X^1,2^		X^2^	X^1,2^
Group Session Scale [65]			X³		X³		
Intervention Delivery Type (Adapted from [64]				X^1^		X^2^	
Intervention Content (Adapted from [64]				X^1^		X^2^	
Individual Qualitative Exit-Interview				X^1^		X^2^	
Implementation Survey (Likert-scale and open-ended items, per [63].)				X^1^		X^2^	
Weekly Facilitator Check-in Questionnaire			X³		X³		

X¹ - Participants who received The CONNECT Program first, X² - Participants who received The CONNECT Program second (after waitlist), X³ - Weekly administration during the CONNECT Program sessions only; not completed during control condition.

Risks of the trial are minimal but may include psychological and emotional discomfort when discussing topics like social isolation, loneliness or mental health. Facilitators monitor participants during sessions and respond to distress following a structured safety protocol. A mental health resource list is provided to all participants, and those in the control condition continue to receive service as usual.

The trial has a target recruitment of 128 participants, with 64 in each delivery group (videoconference and telephone), based on a power calculation with Cohen effect of 0.5 alpha = 0.05 and power 80%. The effect size of 0.5 is based on our pilot data and is consistent with other similar intervention studies in older adults (e.g., [66]).

Participants will be recruited through the four partnering community organizations, with local community partners overseeing outreach. Each site will run two groups per delivery mode (videoconference and phone), with approximately 32 participants recruited per site. To support both recruitment and retention, participants will receive a $25 honorarium (e-gift card or mailed) after completing each of the three assessment points baseline, post-intervention, and follow-up – for a total of $75. An additional $25 honorarium will be provided to participants who are selected at random for qualitative interviews after participating in The CONNECT Program (n = 40; 10 per site).

### Assignment of interventions

Randomization will be conducted by a non-affiliated researcher at the Centre for Healthcare Innovation (CHI) using a secure computerized tool. We are using restricted randomization to keep group sizes balanced within each site. Details about blocking and sequence structure are recorded separately and will not be shared with staff involved in recruitment or group assignment prior to randomization. Each participant will be assigned a unique ID and randomized into one of two sequences: The CONNECT Program first or Community Programming as Usual first. Participants will be randomized prior to enrollment. The research coordinator will be aware of group assignment after randomization to manage scheduling and follow-up; however, outcome assessor and outcome analysts will remain blinded and only work with de-identified data. Group assignment will not be disclosed to the outcome assessor or analysts, and all outcome data will be labeled using participant ID codes without identifying group allocation. Due to the nature of the behavioral interventions, neither participants nor facilitators will be blinded. We do not expect unblinding to be necessary for the assessor or analysts.

If participants are unable to self-administer via a secure link then measures will be administered verbally by a trained research assistant via telephone or university-managed Zoom and responses input on the behalf of a participant. To support data quality, assessors will be trained in standardized procedures, and data will be entered directly into REDCap, which includes built-in range checks. Duplicate entries and inconsistencies will be identified during routine data cleaning. Each participant will be assigned a unique ID number to ensure confidentiality. All data will be stored on secure University of Manitoba (UM) REDCap servers and transferred to a password-protected UM OneDrive folder in accordance with the data transfer agreement, only the research team will have access to identified data.

To promote retention, participants will receive reminders for each assessment and session, along with technical assistance where needed. In cases where participants discontinue or miss sessions, they will still be invited to complete follow-up assessments, including key outcome measures where possible.

Analysis of the primary and secondary outcomes will be conducted using linear mixed-effects models to account for repeated measures and the crossover design. Changes in psychological flexibility, loneliness, social isolation, emotional support, anxiety, depression, and mental health literacy will be compared between The CONNECT Program and control conditions using repeated measures analyses, adjusting for delivery sequence and baseline values. For descriptive comparisons, both paired t-tests and repeated measures ANOVA may be used. Adverse events and participant distress will be recorded; however, harms will not be systematically evaluated. All analyses will be performed on a per-protocol basis, including only participants who complete both stages. Sensitivity analyses may include a larger sample, if applicable. Missing data will be examined for patterns; if data are missing at random, linear mixed models will be used. For key outcomes with high levels of missingness, multiple imputation or pattern-mixture models will be considered. Subgroup analyses by delivery mode and location will be conducted if sample sizes are sufficient to support interpretation.

### Monitoring

A formal Data Monitoring Committee (DMC) was not impaneled for this trial because The CONNECT Program is a low-risk, non-pharmacological behavioral intervention. In accordance with ethical conduct, the principal investigator and study team will provide monitoring of this study. Interim analyses or stopping guidelines are not planned with this short trial and small number of enrolled participants, who are exposed to little risk. Any negative experiences or unexpected issues (e.g., distress among participants) will be managed by the study team. The study coordinator and principal investigator will oversee the daily administration of the program – recruitment, delivery of interventions, attendance, and data collection. The study team will meet on a regular basis to review the progress of the trial and consider any new issues, which have arisen.

### Ethics

This study has received ethical approval from the University of Manitoba Research Ethics Board (REB #HE2024−0150). Any protocol amendments will be submitted for further review and approval. Any significant protocol modifications (e.g., changes in eligibility criteria, outcomes, or procedures) will be communicated to the REB, research team members, community partners, and participants as appropriate. We will provide two options for consenting, with a preference for the first option where feasible. The first option is to electronically sign the consent form in REDCap. If a participant does not have access to a computer/internet, we will offer the alternative of providing verbal consent for participation in the study. Verbal consent will be obtained via telephone and documented in REDCap. The researcher (research coordinator or research assistant) conducting the consenting process over the phone will note the participant’s full name, the date the consent was given, and their own name, indicating who received the consent. Subsequently, we will mail two copies of the consent form to the participant’s residence for their records. Identifiable information will be kept separate from survey responses and stored on secure, password-protected servers. Data will be retained according to institutional data management policies.

## Discussion

The CONNECT Program is a unique mental health support program aimed at addressing the critical service gaps faced by older adults who are experiencing social isolation, loneliness, and mental health difficulties. While our primary aim with this trial is to assess program impact on key mental health outcomes, a secondary aim is to provide real-world evidence about its implementation in different community-service settings. Also, there are a number of design and other administrative issues that should be considered. Although the hybrid trial design allows for simultaneous assessment of implementation and effectiveness outcomes, it may also complicate data interpretation. Contexts between distinct sites vary, potentially confounding attribution of outcomes to intervention.

The individual-level crossover design increases internal validity but may be affected by carryover effects between treatment periods, even with the relatively short 6-week program and no formal washout. Psychological changes may persist and influence subsequent measurements.

Furthermore, tablets and technical support will be offered, and the technological literacy of adults 55 + might influence their engagement, particularly when it comes to the delivery videoconferencing delivery. This can also affect both the implementation and effectiveness data.

Findings from this study will yield critical insights regarding the effectiveness and implementation of The CONNECT Program in a variety of community settings and delivery modalities. Given the urgent need for accessible interventions for socially isolated older adults, a successful outcome of this trial could pave the way for The CONNECT Program to be disseminated broadly as a tool to combat the epidemic of loneliness and mental health challenges in the aging population.

## Supporting information

S1 File SPIRIT 2025 checklist of items to address in a randomized trial protocol*. (DOCX)

S2 File The TIDieR (Template for Intervention Description and Replication) Checklist*. (DOCX)

S3 File Protocol approval. (PDF)

S4 File Metadata. (DOCX)
