# Supplementary material for: Evaluating and implementing The CONNECT Program—A group-based telehealth intervention to reduce social isolation, loneliness, and mental health symptoms in adults 55+ vs routine community programming: Study protocol for a randomized controlled trial
Source: PLoS One. 2025 Nov 11;20(11):e0336031. doi: 10.1371/journal.pone.0336031 (PMC12604767; doi:10.1371/journal.pone.0336031)
Supplement: S3 File — (PDF) [file pone.0336031.s003.pdf]

Research Ethics and Compliance

Human Ethics - Fort Garry
208-194 Dafoe Road
Winnipeg, MB R3T 2N2
T: 204 474 8872
humanethics@umanitoba.ca

PROTOCOL APPROVAL

Effective: July 22, 2024 Expiry: July 21, 2025

Principal Investigator: Kristin Audrey Alison Reynolds
Protocol Number: HE2024-0150
Protocol Title: *The CONNECT Program: Engaging Community Organizations in the Implementation and Evaluation of a Group Telehealth Mental Health Program for Older Canadians*

Sidney Frankel, Acting Chair, REB1

Research Ethics Board 1 has reviewed and approved the above research. The Human Ethics Office (HEO) is constituted and operates in accordance with the current *Tri-Council Policy Statement: Ethical Conduct for Research Involving Humans*- TCPS 2 (2022).

This approval is subject to the following conditions:

i. Approval is granted for the research and purposes described in the protocol only.

ii. Any changes to the protocol or research materials must be approved by the HEO before implementation.

iii. Any deviations to the research or adverse events must be reported to the HEO immediately through an REB Event.

iv. This approval is valid for one year only. A Renewal Request must be submitted and approved prior to the above expiry date.

v. A Protocol Closure must be submitted to the HEO when the research is complete or if the research is terminated.

vi. The University of Manitoba may request to audit your research documentation to confirm compliance with this approved protocol, and with the UM *Ethics of Research Involving Humans*Ethics of Research Involving Humans policies and procedures.

Research Ethics and Compliance

Human Research Ethics - Fort Garry
66 Chancellors Circle
Winnipeg, MB R3T 2N2
humanethics@umanitoba.ca

RENEWAL APPROVAL

Effective: July 3, 2025 New Expiry: July 21, 2026

Principal Investigator: Kristin Audrey Alison Reynolds
Protocol Number: HE2024-0150
Protocol Title: *The CONNECT Program: Engaging Community Organizations in the Implementation and Evaluation of a Group Telehealth Mental Health Program for Older Canadians*

Office of Human Research Ethics as designated by REB1

Research Ethics Board 1 has reviewed and renewed the above research. The Office of Human Research Ethics (OHRE) is constituted and operates in accordance with the current *Tri-Council Policy Statement: Ethical Conduct for Research Involving Humans- TCPS 2 (2022).*

Please note the following important information about your renewal approval:

i. Any changes to the protocol or research materials must be approved by the OHRE **before implementation.**

ii. Any **deviations** to the research or **adverse events** must be reported to the OHRE immediately through an **REB Event**.

iii. This renewal is valid for **one year only**. A Renewal Request must be submitted and approved prior to the above expiry date.

iv. A **Protocol Closure** must be submitted to the OHRE when the research is complete or if the research is terminated.

2. Summary - Summary

Protocol Title	The CONNECT Program: Engaging Community Organizations in the Implementation and Evaluation of a Group Telehealth Mental Health Program for Older Canadians
Principal Investigator	Kristin Reynolds
Is the Principal Investigator for this project a student or post-doctoral fellow?	No
Primary Department	Psychology
Application Initiated By	Kira Kudar

3. Summary - Purpose of the Research

Select the appropriate REB for review:	REB 1
Is this a study that has already been approved by another Canadian institution?	No
Does this study only involve the use of secondary data?	No
Provide a brief statement about the project written in lay language. Do not provide a technical summary.	The COVID-19 pandemic has significantly impacted individuals aged 55 and older, resulting in higher levels of social isolation, loneliness, anxiety, and depression, which can adversely affect cognitive function, physical health, and overall mental well-being. To address this issue, it is essential to combine efforts aimed at promoting social connections with activities that engage cognitive function and target mental health. From 2019 to 2023, our team developed and piloted a telephone-based group mental health program in Manitoba designed for older adults dealing with loneliness, social isolation, and accompanying mental health issues. This CIHR Project Grant study is a natural progression from our previous research efforts. This research aims to engage community organizations in four Canadian provinces — Manitoba, Saskatchewan, British Columbia, and New Brunswick — in implementing and evaluating The CONNECT Program.
In point form, list the research question(s) and objectives for this research study.	Our specific objectives are to: • 1 - Understand the mental health and service needs of adults ages 55+ as well as community providers across the 4 partnership sites • 2 - Understand the revisions that need to be made to CONNECT Program materials based on the perspectives of adults 55+ and community providers across the 4 partnership sites • 3 - Explore the involvement of partners across the 4 sites and the degree to which they are satisfied with their level of involvement (integrated knowledge translation evaluation) • 4 - Examine the implementation and effectiveness of The CONNECT Program in improving key social and mental health indicators, as compared to service as usual (defined as typical community program participation). Research questions: • 1 - What are the service needs of adults 55+ and community service providers? • 2 - How should the CONNECT program be tailored to meet region-specific needs? • 3 - When compared to service as usual, how much does The CONNECT Program bring additional benefit in key implementation and effectiveness indicators? • 4 - How do telephone and virtual modalities compare in terms of the evaluation of key implementation and effectiveness indicators? • 5 - How do community providers experience their partnership/involvement with the research team, project, and process? • 6 - How do community providers (facilitators) evaluate the CONNECT training before and after the training and after the program delivery?

Describe the research methods.	Prior to implementing the CONNECT program, a focus group will be conducted with community providers (e.g., staff, volunteers, mental health workers of partner organizations) and adults 55+ at each site, in accordance with the prepared focus group protocol. Utilizing an integrated knowledge translation framework proposed by Kothari et al. (2017), this study adopts an implementation effectiveness hybrid design (Landes et al., 2020) coupled with a crossover randomized trial design (RCT; Sibbald & Roberts, 1998) to evaluate the implementation of the CONNECT program being offered by telephone and virtually. To do so, both quantitative and qualitative methodologies will be employed. Following the Proctor approach to implementation evaluation (Proctor et al., 2011), the research will assess eight distinct implementation outcomes through various methods, including participant surveys with both open-ended and Likert-scale items, participant interviews, and a focus group with facilitators. In examining the experience of partnerships with the research team, we will employ a quantitative survey at years 1, 2, 3, and end of year 4 of the project, and a focus group with community providers at year 1 and end of year 4. Facilitators will complete an evaluation that includes standardized questionnaires, open-ended questions, and Likert-scale items at several time points: before the training, immediately after the training, after delivering their first CONNECT group, and through weekly check-ins during the program implementation at their site.
Briefly describe chronologically what the research team will ask participants to do after consent has been obtained. If there are multiple phases of the research, describe each phase.	• 1 - Focus group for community providers. Before initiating the study, the research team will organize a focus group involving the research team and staff, volunteers, mental health workers of partner organizations engaged in the project. The aim is to collect feedback on the effective implementation and delivery of the program, as well as to tailor materials according to the specific regional needs of each site (Manitoba, Saskatchewan, British Columbia, and New Brunswick). • 2 - Interview or Focus group for adults 55+. Before initiating the study, the research team will organize an interview or focus group at each site involving adults 55 years and older who are seeking to better understand their emotions, and to build connections with others and what is important to them (i.e., potential CONNECT program users). The aims are to understand participants' identities, mental health needs, perspectives on the suitability of CONNECT program, and suggestions for implementing the CONNECT program. The focus groups/ interview will provide information on how to effectively tailor and implement the CONNECT program to meet region-specific needs for Manitoba, Saskatchewan, British Columbia, and New Brunswick. • 3 - Facilitator Training and Evaluation. Before program implementation, the research team will conduct CONNECT Program training sessions to prepare community providers from partner organizations to deliver the program. Participation in the training will be evaluated at a few time points: before the training, immediately after, after each group session (a brief weekly check-in), and following program delivery. • 4 - Randomization and assignment. This step will occur after the CONNECT program has been tailored using the feedback obtained from the focus groups. Eligible adults 55+ will be recruited to participate in the CONNECT program at each site. After obtaining consent from recruited participants, they will be randomly assigned within community sites to

intervention or control (community organization participation as usual). For participants who are randomized into intervention they will be randomized into either the Phone or Virtual condition. Each site will implement either the Zoom then Phone or Phone then virtual delivery mode sequence. • 5 - Phone-based or virtual therapeutic sessions. Participants will attend six therapeutic sessions, which will be held weekly and last 90 minutes each. These sessions will include structured activities aimed at enhancing their mental well-being. Additionally, participants will be given weekly homework assignments to practice the therapeutic skills learned during the sessions. Workbooks summarizing session information and at-home activities will be provided in a format preferred by participants. These sessions will be conducted over phone or virtually (e.g., Zoom). After each session, the study coordinator will contact participants individually by telephone for a 5-minute survey with 4 questions regarding how they felt during the group session that day. • 6 - Implementation and Effectiveness Data Collection and Evaluation. Participants will undergo evaluation three times: first, before starting their initial treatment period; second, following the treatment period; and finally, 6-month follow-up. Data collection will span six months at each site, consisting of a three-month telephone intervention period offering two 6-week programs, followed by a three-month virtual intervention period offering two additional 6-week programs. Measurements will be taken at three time-points: baseline, post-CONNECT, and 6-month follow-up, in line with ACT treatment literature. • 7 - Completion of intervention. All participants will receive the intervention before the trial concludes, ensuring equitable treatment for everyone involved. • 8 - Qualitative interviews. Upon completion of the intervention, qualitative interviews will be conducted with interested participants to delve deeper into their experiences and perceptions, providing valuable insights into the effectiveness of the program. Purposive sampling will be employed to ensure that we have heard from diverse perspectives including age, gender, culture, ability. • 9 - Additionally, the Integrated Knowledge component will be included into the research, meaning that the experiences of community providers will be investigated through pre- and post-program focus groups, and surveys (Y1, Y2, Y3, Y4), as well as during partner meetings (meeting agendas and minutes).

Where will the study take place?	Four centers: A&O: Support Services for Older adults in Manitoba, Senior Citizens Assistance Program (SCAP) in Saskatchewan, Brella Community Services Society in British Columbia, and New Brunswick Community sites. Screening will occur virtually either over the phone or Zadarma - this is VoIP provider (telephone communication that allows us to obtain work numbers for our team, as well as toll-free numbers for participants' convenience). Focus groups will be facilitated virtually via UM Zoom Professional. The CONNECT program will be offered virtually (e.g., Webex or Zoom) and via phone (generated through Webex or Zoom). Focus groups will be conducted virtually or by phone/audio. Questionnaires will be completed either virtually via REDCap, over Zoom with screen sharing or over the telephone. Interviews will be conducted either virtually via Zoom Professional or by audio/telephone. Data collection and analysis will be conducted at the University of Manitoba.

How much time will study participants be expected to dedicate to the research?	We have 2 main samples in this work, adults 55+ and community providers (e.g., staff, volunteers, mental health workers at the 4 sites). Adults 55+ (Intervention and Control Conditions) 1st part (understanding service needs and program revisions)* not all participants will complete • Screening (eligibility for interview or focus group study) - 30 min • 1 interview or focus group (understanding service needs and program revisions) - 60-90 minutes • Questionnaires - 25 min 2nd part (examine the implementation and effectiveness of The CONNECT Program) • Screening (eligibility for CONNECT Program participation) - 15 min • CONNECT Program Participation *timing of this will differ for control condition participants and not all participants may be interested in completing this. The intervention consists of 6 weekly, 90-minute group sessions • Questionnaire Evaluation - 5 min survey after each session, and participants will undergo evaluation three times, each lasting approximately 45 minutes (baseline, 6-month follow-up) and 60 minutes (post-CONNECT). 3rd part *not all participants will complete • Qualitative Interview - 60 min • Total Anticipated Participation Time: understanding service needs and program revisions – 145 min, CONNECT program participation and evaluation – 735 min, interview – 60 min. Overall: 940 min (15 hours and 40 minutes) over 4 years. Community providers: 1st part (understanding service needs and program revisions) • 1 - Focus Group - 90 minutes • 2 - Questionnaire - 60 min 2nd part (explore the involvement of partners across the 4 sites) • 3 - Questionnaire at each year (Y1, Y2, Y3, Y4; understanding partnerships and involvement) - 20 minutes per questionnaire • 4 - Focus Groups (Y1 and Y4; un understanding partnerships and involvement) - 90 minutes • 5 - Regular Team Meetings - (approximately 4 meetings per year, 60 minutes per meeting) • 6 - Training: 2 sessions × 180 minutes = 360 minutes (6 hours), Training-related questionnaires (pre-, post-, and follow-up): 3 surveys × 20 minutes = 60 minutes (1 hour) • Weekly check-in questionnaires during program delivery: 4 groups × 6 weeks × 10 minutes = 240 minutes (4 hours), post-program focus group participation (1 hour). • 7 - Group facilitation: 4 groups × 6 sessions × 90 minutes = 2,160 minutes (36 hours) Note: Multiple facilitators will be trained per site, allowing for co-facilitation or rotation after completing at least one group. • Total Anticipated Participation Time: 3530 min (58 hours, 50 min) over 4 four years.

4. Summary - Research

Type of Research (select all that apply):	Faculty Research, Master's Thesis, Honour's Thesis
Type of Study (select all that apply):	Randomized Control Trial, Survey/Questionnaire, Focus Group/Interview, Other
Other Type of Study	Implementation-effectiveness hybrid design and a crossover randomized trial design

5. Summary - General Questions

Does the study involve adult participants who are not legally or practically able to give their valid consent to participate?	No
Does the study involve participants who are under the age of 18?	No
Are participants from a population that may be marginalized or vulnerable in the context of research?	No
Does this research include the use of personal health information?	Yes

The Manitoba Personal Health Information Act (PHIA) outlines responsibilities of researchers to ensure safeguards that will protect personal health information. PHIA requires that all research team members who handle or are exposed to personal health information take the University of Manitoba's PHIA orientationThe University of Manitoba's PHIA orientation and sign a pledge of confidentiality that acknowledges that they are bound by written policy and procedures. The University of Manitoba PHIA orientation and pledge signing must be completed by all research team members.

Indicate provisions that will be made to comply with PHIA.	All research team members completed the PHIA training and uploaded their signed pledges of confidentiality or certificates.
Does this study use deception (e.g., will participants be intentionally misled about the studys purpose, their own performance, or other features)?	No
Will the majority of participants identify as First Nations, Inuit, and/or Metis?	No
Will the analysis of the research results use First Nations, Inuit, and/or Metis identity as a variable?	No
Will the interpretation of research results refer to First Nations, Inuit, and/or Metis people, language, history or culture?	No
Will participants be given the choice to waive their anonymity?	No
Does this study require approval from another organization?	No

Will this study include involvement or recruitment from a specific group or organization?	Yes
Will permission be required to conduct the study outside the University of Manitoba?	No

7. Participants - Participants

How many participants do you expect to recruit?	Community Providers • Objectives 1 and 2 - Within each site, approximately 4 community providers will be recruited, with N=16 in total for focus group. • Objective 3 - Within each site, approximately 4 community providers will be recruited, with N=16 in total for IKT component (focus groups Y1 and Y4, partner meetings, surveys Y1, Y2, Y,3, Y4). Training and Facilitation. A total of 8 to 16 potential facilitators will be recruited in the four sites. At each community site, 2 to 4 facilitators will be invited to participate in the CONNECT Program training and subsequently deliver the program locally. Adults 55+ • Objectives 1 and 2 - Within each site, approximately 4-8 adults 55+ will be recruited. At maximum, this will result in a total of n = 32 adults 55+ across the four sites. • Objective 4 - Within each site, approximately 8 participants will be involved in each condition (following best practice guidelines for group psychotherapy), resulting in a total number of n = 32 participants in BC, MB, NB, SK and N = 128 participants in total across the four sites. At each site participants will be recruited and randomly assigned to one of two within-participant treatment sequences: Intervention-Control or Control-Intervention.
What are the inclusion criteria to participate in the study?	Community providers: • Employment or volunteering in a partnering organization. • Specialization in mental health (psychologist, psychiatrist, social worker, etc.). • Age 18 years or older. • Willingness and ability to participate in the focus group and adhere to the scheduled timing. • Ability to express thoughts and views on discussed topics in English. Adults 55+ • The age eligibility to 55 years and older. • Participants can communicate (talk and read) in English. • Participants should manage changes in hearing/vision impairment such that they can attend to group conversations, and self-report challenges related to loneliness, social isolation, and co-occurring mental health problems (anxiety or depressive symptoms).
What are the exclusion criteria?	Community providers: • Lack of relevant work experience or affiliation with partner organization. • Lack of interest in the discussed topic. Adults 55+ • Can't communicate (talk and read) in English.
Will the participants in your study be UNAWARE that they are participants?	No
Will information about the participants be obtained from sources other than the participants (e.g., obtaining contact information from public sources, parents providing data on behalf of their child)?	No
Will participants receive any compensation for participating (e.g. honorarium, course credit, food, parking)?	Yes

8. Participants - Compensation

What is the compensation for participating?	Community providers • #ommunity provider focus group will also include a background survey. Participants in the study will include staff, volunteers, and mental health workers from partner organizations involved in the project. These participants will receive either an e-gift card or a mailed gift card valued at $20 for survey completion and $25 for participation in the focus group. • Partner meetings (approximately 4 meetings per year), honorarium $25 per meeting. • Focus groups with community providers (IKT component) Pre-Program $25 honorarium, Post-Program $25 honorarium. • Questionnaires for community providers Y1, Y2, Y3, Y4, honorarium $20 per questionnaire. Also centres will be reimbursed for poster postage. • CONNECT program training and facilitation - Community providers will receive a $150 gift card for participating in the 6-hour CONNECT Program training. Additionally, they will receive a $50 gift card for completing the training-related surveys (pre-training, post-training, weekly session check-ins, and follow-up after facilitation). 25$ for participation in the post-delivery focus group. Program Facilitation - facilitators will be compensated $40 per group session facilitated. Compensation for facilitation will be distributed at the end of the delivery. Adults 55+ • Interview or focus group: These participants will receive an e-gift card or mailed gift card valued at $50. • Intervention: Participants (N = 128) will receive honorariums (e-gift card or mailed gift card valued at $25) for participating in the quantitative baseline, post-CONNECT, and 6-month follow-up assessments ($75 total). Participants will also receive a $25 honorarium if they are selected to complete the qualitative interview at post-CONNECT assessment N = 40 (10 per 4 provincial sites).
Provide justification for these compensation arrangements.	Our compensation arrangements serve various important purposes. It acknowledges people's efforts, encourages them to join in, ensures fairness, and follows ethical guidelines. The payment we offer is modest. This is to make sure that e-gift card isn't the only reason people get involved. We want to highlight that participating is voluntary and that contributing to the growth of knowledge is really meaningful.
When will participants receive their compensation?	• Community providers will receive their compensation at the start of each research activity such as initial focus group (objective 1 and 3), and at the start of IKT component activities such as focus groups Y1, Y4; surveys Y1, Y2, Y3, Y4 and partner team meetings. At the start of the training and before completing the training-related evaluations and focus group participation; compensation for facilitation will be distributed at the end of the delivery. • Adults 55+ will receive their compensation at the start of the focus group and each assessment session (i.e., baseline, post-CONNECT, and 6-month follow-up assessments). Also, compensation will be provided to the 40 participants selected for qualitative interviews at the start of post-CONNECT assessment.
Participants must be able to keep their compensation if they withdraw from the study.	Participants who decide to withdraw from the study will still be allowed to keep their compensation. This ensures that

Describe how participants will be compensated if they withdraw from the study.	they are not financially penalized for their decision. Upon withdrawal, participants will be informed that they can retain the compensation they have already received.

9. Participants - Recruitment

Describe how prospective participants will be identified, who will contact them, and the process of doing so.

• Community providers: Prospective participants will be identified via self-referral, and will be asked to reach out to the study team via telephone or email. We will send emails to the providers of partner organizations with information about the focus group, IKT component activities, and the Training and Facilitation component, and the conditions of participation. We will also attach an electronic poster and ask them to share it with their staff, volunteers, and mental health professionals in those organizations by physically posting the advertisement in their centres and by sending it through mail and email. If sites have websites, we will also ask them to post the advertisement on their website. We will leave our study contact information (telephone, email) on the recruitment material for potential participants to reach out to us. • Adults 55+ interview/focus group and CONNECT program: Prospective participants will be identified via self-referral and will be asked to reach out to the study team via telephone or email. We will send emails to the providers of partner organizations with information about the focus group/interview and the CONNECT program groups. We will also attach an electronic poster and ask them to share it with members of their organizations by physically posting the advertisement in their centres and by sending it through mail and email. If sites have websites, we will also ask them to post the advertisement on their website. We will also share electronically an infographic poster with our partners, so they can share with potential participants (adults 55+) an overview of the CONNECT program, its goals, format, and what each phone session will look like. Centres will be reimbursed for postage. We will leave our study contact information (telephone, email) on the recruitment materials for potential participants to reach out to us.

Attach copies of all material that will be posted/given/read to participants and/or third parties (e.g., email and telephone scripts, social media posts, captions, posters, letters).

Type	Name	Document
Recruitment Documents	Community providers FG poster	Community providers FG poster.pdf
Recruitment Documents	IKT poster	IKT poster.pdf
Recruitment Documents	Adults 55+ FG poster	Adults 55+ FG poster - V2.pdf
Recruitment Documents	Adults 55+ CONNECT poster	Adults 55+ CONNECT poster.pdf
Recruitment Documents	Community providers FG recruitment email	Community providers FG recruitment email.docx
Recruitment Documents	IKT recruitment email	IKT recruitment email.docx

10. Consent - Informed Consent Process

Describe the consent process including how consent will be obtained.	Consent will precede data collection. Participants will receive detailed explanations of the study, followed by the opportunity to provide consent. • Community providers - e-signature in an electronic consent form in REDCap. • Adults 55+ - We will provide two options for consenting and signing the oath of confidentiality, with a preference for a digital format, where feasible. The first option is to electronically sign the consent form and oath of confidentiality in REDCap. If a participant does not have access to a computer/ internet, we will offer the alternative of providing verbal consent for participation in the study. Verbal consent will be obtained via telephone. Verbal consenting will be recorded in REDCap. The researcher conducting the consenting process over the phone will note the participant's full name, the date the consent/oath of confidentiality was given, and their own name, indicating who received the consent. Subsequently, we will mail two copies of the consent form to the participant's residence for their records.

Attach all consent forms as individual documents.

Type	Name	Document
Consent Documents	Community providers FG Consent form	Community providers FG Consent V3.docx
Consent Documents	IKT Consent form	IKT Consent form V3.docx
Consent Documents	Adults 55+ FG consent form	Adults 55+ FG consent form - V3.docx
Consent Documents	Adults 55+ CONNECT Consent form	Adults 55+ CONNECT Consent form V5.docx
Consent Documents	Community Providers Training and Facilitation - Consent Form	Community Providers Training and Facilitation - Consent Form.docx

11. Data - Confidentiality

Privacy refers to a participants right to be free from intrusion or interference by others.

Confidentiality refers to the researcher's/research team's obligation to safeguard entrusted information.

Are there conditions in which privacy or confidentiality cannot be guaranteed (e.g., a group setting, or when third parties might be aware of participant involvement, interviews in public spaces)?	Yes
Please explain the precautions you will take to protect privacy and confidentiality.	Since the program will be delivered in group settings, including CONNECT and focus group sessions, participants will be notified in advance in the consent form that confidentiality cannot be guaranteed for spoken information shared during the group sessions. Participants will be asked to be mindful of this when sharing any private or sensitive personal information with the group. Additionally, they will be reminded to respect the rights to privacy and confidentiality of other group members and not to disclose any information shared within the context of the group sessions. Furthermore, all group members will be required to sign an oath of confidentiality in order to participate in the CONNECT program groups (Adults 55+ CONNECT) and Adults 55+ FG.

12. Data - Data

Please review the different types of information researchers may seek to collect, use, share and access based on the TCPS 2, Chapter 5

Anonymous information – the information never had identifiers associated with it (e.g., anonymous surveys) and risk of identification of individuals is low or very low.

Anonymized information – the information is irrevocably stripped of direct identifiers, a code is not kept to allow future re-linkage, and risk of re-identification of individuals from remaining indirect identifiers is low or very low.

Directly identifying information – the information identifies a specific individual through direct identifiers (e.g., name, social insurance number, personal health number).

Indirectly identifying information – the information can reasonably be expected to identify an individual through a combination of indirect identifiers (e.g., date of birth, place of residence or unique personal characteristic).

Coded information – direct identifiers are removed from the information and replaced with a code. Depending on access to the code, it may be possible to re-identify specific participants (e.g., the principal investigator retains a list that links the participants' code names with their actual name so data can be re-linked if necessary).

Will this study include a survey or questionnaire?	Yes
How will the survey/questionnaire be administered?	A questionnaires will be administered either via telephone or UM Zoom by a research coordinator or research assistant following a pre-prepared script, these raw data will be stored on in the REDCap server, or completed by participants online through a secure REDCap link sent to their email. Participants (Adults 55+) may receive up to three email/ call reminders, each within 48-hour intervals, to complete baseline, post-program, follow-up, or weekly post-session questionnaires. A survey for the staff, volunteers, mental health workers of partner organizations as part of a focus group and IKT component will be also conducted online using the REDCap. The Community Providers FG Feedback Survey, where participants will provide written feedback on the materials, will be sent by email and returned via email in a Word doc file.
Describe the type(s) of data to be collected: anonymous, anonymized, coded, indirectly identifiable and/or directly identifiable.	The data obtained from surveys/questionnaires will be coded, meaning that once a participant signs the consent form and is assigned to a group, their personal details will be removed and replaced with a code. Links to participant information will be stored in a master list, which will be maintained according to the guidelines of the Extreme Risk Level Data Storage Guidelines. Any data collected (i.e. online surveys) will be identified by the unique code or participant's ID only. Only the organization's name will be used in the Community

Providers FG Feedback Survey as an indirect identifier to ensure the feedback is linked to the correct revisions.

Do you intend to provide participants feedback based on the survey/questionnaire results?	No
Where will the survey/questionnaire data be stored in the short term and long term?	This data will be stored on REDCap Survey Server and survey data will be periodically transferred to a secure file location (UM One Drive account) (as stated in the REDCap data transfer agreement form). The Community Providers FG Feedback Survey responses, after being received via email from participants, will be transferred for storage in the UM OneDrive folder.
How long will you keep the data? If you intend to destroy the data, when (MMYY)? Provide justification if data will be kept indefinitely.	This data will be kept for one year after the conclusion of the study until the results are published (approximately 7 years: March, 2031). Keeping this data until March 2031 will ensures that we can fully analyze data, share our findings with participants and stakeholders, and disseminate the results within the research community.

Attach survey(s)/questionnaire(s) as individual documents.

Type	Name	Document
Survey/Questionnaire Document(s)	Community providers FG Background Questionnaire	Community providers FG Background Questionnaire.docx
Survey/Questionnaire Document(s)	IKT Questionnaire (Y1, Y2, Y3)	IKT Questionnaire (Y1, Y2, Y3).docx
Survey/Questionnaire Document(s)	IKT PEIRS - Patient Engagement In Research Scale	IKT PEIRS - Patient Engagement In Research Scale.pdf
Survey/Questionnaire Document(s)	IKT Questionnaire (Y4)	IKT Questionnaire (Y4).docx
Survey/Questionnaire Document(s)	Adults 55+ FG questionnaires	Adults 55+ FG questionnaires - V2.docx
Survey/Questionnaire Document(s)	Adults 55+ CONNECT Baseline-follow up Questionnaire	Adults 55+ CONNECT Baseline-follow up Questionnaire.docx
Survey/Questionnaire Document(s)	Adults 55+ CONNECT Post-program Questionnaire	Adults 55+ CONNECT Post-program Questionnaire.docx
Survey/Questionnaire Document(s)	Adults 55+ CONNECT Group Session Scale	Adults 55+ CONNECT Group Session Scale.docx
Survey/Questionnaire Document(s)	Community Providers FG Feedback Survey	Community Providers FG Feedback Survey.docx
Survey/Questionnaire Document(s)	Community Providers Training and Facilitation - pre, post, follow-up surveys	Community Providers Training and Facilitation - pre, post, follow-up surveys.docx

Type	Name	Document
Survey/Questionnaire Document(s)	Community Providers Training and Facilitation - Weekly facilitator check-in questionnaire	Community Providers Training and Facilitation - Weekly facilitator check-in questionnaire.docx

Does this study involve interviews?	Yes
Who will conduct the interview?	Principal Investigator, Research Coordinator and Research assistants.
Will any individual(s) other than the research personnel be present during the interview?	No
Will these interviews involve audio recording or, video recording?	Yes
List the procedures that will be recorded (e.g., obtaining consent, preamble, interview itself).	The conversation between the research staff and the participant will be audio recorded during the interview questions. Closed captions will be used to facilitate the subsequent transcription process.
State the purpose of recording.	For transcribing the collected qualitative data.
Will participants be permitted to review, edit, and/or erase the recording?	No
Where will the recordings be stored? How will you maintain participant confidentiality?	UM Zoom audio recordings will be stored on UM OneDrive or UM-managed computers. Additionally, if participants do not have access to a computer and the internet, we will use cell phone audio recording using Zadarma VoIP service. Immediately after recording, the file will be saved on UM OneDrive or UM-managed computer. We acknowledge that recordings should be transferred to the UM-approved primary storage location at the earliest opportunity and no later than one week after recording.
If the recordings are transcribed, what will happen to the recording post-transcription? Provide the date the recordings will be destroyed (MMYY) or provide a justification for keeping the recordings indefinitely.	The recordings of the interviews will be destroyed immediately after transcription.
Who will transcribe the recordings? Indicate if the recordings will be transcribed manually or identify the transcription service used.	Principal Investigator, Research Coordinator and Research assistants. Also, we plan to use Trint Qualitative Transcription Software, we will contact the company and ask that the data be destroyed after transcription is complete.
Will the transcripts be anonymized, coded, or identifiable? Please explain	The transcripts will be coded - once the participant has signed the consent form and been assigned to a group, their direct identifiers will be removed from the information and replaced with a code.

Where will the transcripts be stored?	Coded transcripts will be stored on UM OneDrive/or on UM managed computers with password protection.
What will happen to the transcripts? Provide the date the transcripts will be destroyed (MMYY) or provide a justification for keeping the transcripts indefinitely.	These transcripts will be kept for one year after the conclusion of the study until the results are published (approximately 7 years: March, 2031). Keeping this data until March 2031 will ensures that we can fully analyze data, share our findings with participants and stakeholders, and disseminate the results within the research community.

Attach interview questions as individual documents.

Type	Name	Document
Interview Document(s)	Adults 55+ CONNECT Individual Qualitative Exit-Interview	Adults 55+ CONNECT Individual Qualitative Exit-Interview.docx
Interview Document(s)	Adults 55+ FG Interview protocol	Adults 55+ FG Interview protocol.docx

Does this study involve focus groups?	Yes
Who will conduct the focus groups?	Principal Investigator, Research Coordinator and Research Assistants
Will any individual(s) other than the research personnel be present during the focus groups?	Yes
Identify and describe who will be present (e.g., facilitators, translators, support persons, Elders).	Staff, volunteers and geriatric mental health professionals of the partner organizations in four sites of Canada.
Will these interviews involve audio recording or, video recording?	Yes
List the procedures that will be recorded (e.g., obtaining consent, preamble, focus group itself).	The conversation between the participants of the virtual focus group will be captured via UM Zoom recording. Closed captions will be used to facilitate the subsequent transcription process.
State the purpose of recording.	For transcribing the collected qualitative data.
Will participants be permitted to review, edit, and/or erase the recording?	No
Where will the recordings be stored? How will you maintain participant confidentiality?	UM Zoom recordings will be stored on UM OneDrive or on UM-managed password-protected computers.
If the recordings are transcribed, what will happen to the recordings post-transcription? Provide the date the recordings will be	The UM Zoom recording of the virtual focus group conversation will be destroyed immediately after transcription.

destroyed (MMYY) or provide a justification for keeping the recordings indefinitely.

Who will transcribe the recordings? Indicate if the recordings will be transcribed manually or identify the transcription service used.

Principal Investigator, Research Coordinator and Research Assistants. Also, we plan to use Trint Qualitative Transcription Software, we will contact the company and ask that the data be destroyed after transcription is complete.

Will the transcripts be anonymized, coded, or identifiable? Please explain

Participants will identify themselves by their first names during the focus group session. Subsequently, once the focus group is completed, their names will be replaced with pseudonyms or numbers (coded) to ensure anonymity in the transcripts.

Where will the transcripts be stored?

Coded transcripts will be stored on UM OneDrive/ or UM managed password-protected computers.

What will happen to the transcripts? Provide the date the transcripts will be destroyed (MMYY) or provide a justification for keeping the transcripts indefinitely.

These transcripts will be kept for one year after the conclusion of the study until the results are published (approximately 7 years: March, 2031). Keeping this data until March 2031 will ensures that we can fully analyze data, share our findings with participants and stakeholders, and disseminate the results within the research community.

What will happen to the recordings and/or transcripts if one participant (or more) decides to withdraw?

We will include parts of the recording from consenting participants or edit the full recording to remove information from the individual who has withdrawn. We will also remove specific participant quotes from a transcript. We will inform participants during the consent process that, due to the audio and feedback being combined with those of other participants in the focus group, some parts of the recording or the entire recording cannot be completely destroyed.

Attach focus group questions and guide as individual documents.

Type	Name	Document
Focus Group Document(s)	Community providers FG Focus Group Protocol	Community providers FG Focus Group Protocol.docx
Focus Group Document(s)	IKT Focus group protocol (Y1)	IKT Focus group protocol (Y1).docx
Focus Group Document(s)	IKT Focus group protocol (Y4)	IKT Focus group protocol (Y4).docx
Focus Group Document(s)	Adults 55+ FG focus group protocol	Adults 55+ FG protocol - V2.docx
Focus Group Document(s)	Community Providers Training and Facilitation - Focus group	Community Providers Training and Facilitation - Focus group.docx

Does your study involve observations?

No

Are there any other data mediums that are not listed above? This includes paper consent forms, paper assent forms, photos, participant

Yes

ID keys/codebooks, handwritten notes, observation notes, log files, articifacts (e.g. notes, gifts, cards).

In addition to any data described above, select other data you may create, collect, access, store or share during or related to this study and complete the table below.

Data Medium	Participant ID key
Describe the type(s) of data to be collected: anonymous, anonymized, coded, indirectly identifiable and/or directly identifiable.	Directly identifiable data.
Where will the data be stored? Include information on both physical and electronic copies.	Data will be stored on UM One Drive and will be encrypted. Access restricted to limited number of research team members only (Principal Investigator and Research Coordinator).
How long will you keep the data? If you intend to destroy the data, when (MMYY)? Provide justification if the data will be kept indefinitely.	The data will be destroyed once we finish the research and share the results with the participants (approximately 7 years: March, 2031). Keeping this data until March 2031 will ensures that we can fully analyze data, share our findings with participants and stakeholders, and disseminate the results within the research community.
Data Medium	Other
Describe the type(s) of data to be collected: anonymous, anonymized, coded, indirectly identifiable and/or directly identifiable.	Minutes and agenda from the partner meetings. Anonymized data.
Where will the data be stored? Include information on both physical and electronic copies.	Will be stored on UM One Drive and available for all research team.
How long will you keep the data? If you intend to destroy the data, when (MMYY)? Provide justification if the data will be kept indefinitely.	The data will be destroyed once we finish the research and share the results with the participants (approximately 7 years: March, 2031). Keeping this data until March 2031 will ensures that we can fully analyze data, share our findings with participants and stakeholders, and disseminate the results within the research community.
Data Medium	Honorarium receipt forms
Describe the type(s) of data to be collected: anonymous, anonymized, coded, indirectly identifiable and/or directly identifiable.	Directly identifiable data.
Where will the data be stored? Include information on both physical and electronic copies.	Data will be stored on UM One Drive and will be encrypted. Access restricted to limited number of research team members only (Principal Investigator and Research Coordinator).

How long will you keep the data? If you intend to destroy the data, when (MMYY)? Provide justification if the data will be kept indefinitely.	The data will be destroyed once we finish the research and share the results with the participants (approximately 7 years: March, 2031). Keeping this data until March 2031 will ensures that we can fully analyze data, share our findings with participants and stakeholders, and disseminate the results within the research community.
Data Medium	Other
Describe the type(s) of data to be collected: anonymous, anonymized, coded, indirectly identifiable and/or directly identifiable.	List for future contact. Directly identifiable data.
Where will the data be stored? Include information on both physical and electronic copies.	Data will be stored on UM One Drive and will be encrypted. Access restricted to limited number of research team members only (Principal Investigator, Research Coordinator, Research Assistant - Inga Christianson).
How long will you keep the data? If you intend to destroy the data, when (MMYY)? Provide justification if the data will be kept indefinitely.	The data will be destroyed once we finish the research and share the results with the participants (approximately 7 years: March, 2031). Keeping this data until March 2031 will ensures that we can fully analyze data, share our findings with participants and stakeholders, and disseminate the results within the research community.
Data Medium	Other
Describe the type(s) of data to be collected: anonymous, anonymized, coded, indirectly identifiable and/or directly identifiable.	Digital consent forms. Directly identifiable data.
Where will the data be stored? Include information on both physical and electronic copies.	Data will be stored on UM One Drive and will be encrypted. Access restricted to limited number of research team members only (Principal Investigator and Research Coordinator).
How long will you keep the data? If you intend to destroy the data, when (MMYY)? Provide justification if the data will be kept indefinitely.	The data will be destroyed once we finish the research and share the results with the participants (approximately 7 years: March, 2031). Keeping this data until March 2031 will ensures that we can fully analyze data, share our findings with participants and stakeholders, and disseminate the results within the research community.
Data Medium	Other
Describe the type(s) of data to be collected: anonymous, anonymized, coded, indirectly identifiable and/or directly identifiable.	Screening notes. Directly identifiable data.
Where will the data be stored? Include information on both physical and electronic copies.	Data will be stored in password-protected file on password-protected UM computer.

How long will you keep the data? If you intend to destroy the data, when (MMYY)? Provide justification if the data will be kept indefinitely.	The data will be destroyed immediately once we determine the participant's eligibility.
Data Medium	Other
Describe the type(s) of data to be collected: anonymous, anonymized, coded, indirectly identifiable and/or directly identifiable.	Email communication. Directly identifiable data.
Where will the data be stored? Include information on both physical and electronic copies.	Email communication will be made through UM Microsoft Outlook.Emails will remain on the UM Outlook server and will not be saved/retained elsewhere.
How long will you keep the data? If you intend to destroy the data, when (MMYY)? Provide justification if the data will be kept indefinitely.	Emails will be deleted at the end of the project data collection period (when correspondence with participants is complete).

13. Data - Data Transfer

Will data be transferred from one site to another? This includes data collected in the field or off-site.	No
Will data be shared/transferred between research team members (e.g., student PIs/ advisors, research assistant/PI, PI/co-Is)?	Yes
Please describe in detail how the data will be transferred. What identifiable data will be transferred? How and where will it be stored? What safeguards will be used to protect the data during transfer and storage?	• The Principal Investigator and Research Coordinator have access to a master list of participants and their codes. Encrypted files with identifiable data will be shared between PI and RC through UM OneDrive. PI and RC will share links with each other instead of data files to minimize risk. • All research team members will have access to virtual documents that contain already coded or anonymized information. These coded data will be stored on UM OneDrive or REDCap Survey Server. The first survey (collecting only demographic information) will be administered to all participants and the collected data will be exported out from the survey server and stored on the UM OneDrive. Next, all project data housed in the survey server will be erased before administering the health surveys. After completing the health surveys, project data will be export to the UM OneDrive, erase all the project data and finally, the project will be archived. • Encrypted and password-protected files will be shared via UM email and UM OneDrive. Team members will share links instead of data files to minimize risk.
Will the data be archived or made accessible to the public and/or other researchers?	No

14. Risks/Benefits - Benefits

What are the expected benefits of the research?	The expected benefits of the research include contributing to a deeper understanding of the topic, potentially leading to improved interventions or treatments for the target adults 55+ population. Additionally, participation in the research may provide personal development opportunities for participants. Overall, the research aims to contribute positively to knowledge, practice, and the well-being of individuals and society.
What are the indirect benefits for participants participating in the research?	Participants may experience a sense of satisfaction from knowing that they are helping to make a positive impact by contributing to the advancement of knowledge in the field that could ultimately benefit others.
What are the direct benefits for participants participating in the research?	The direct benefits for participants in the research may include access to the CONNECT program that could improve their mental well-being or address their needs. For community providers, the direct benefits include networking opportunities and skill development, enhancing their research skills.

15. Risks/Benefits - Risks

What are the risks (psychological, physical, emotional, social, legal, economic, or political) to participants and others if applicable.	The risk of participating in the trial is minimal. Psychological and emotional risks.
Explain the steps that will be taken to reduce or mitigate any actual harm to participants (e.g., providing a list of resources, providing a safe space, having an Elder or counselor available).	Psychological and emotional - discussing and reading about problems such as isolation, loneliness, anxiety, and depression may carry with it the risk of experiencing increased emotional distress. Participants within the control condition will have access to service as usual, and a mental health resource list will be provided to all participants who document study consent, in the case that additional needs arise.
Is there a possibility that abuse of children or persons in care might be discovered in the course of the study?	Yes
Current laws require that allegations of certain offenses against children or persons in care be reported to legal authorities. Indicate the provisions that will be made for complying with the law.	In the consent form, it is explained that confidentiality may be breached in certain circumstances to comply with legal obligations. For instance, suspected or unreported cases of child abuse will be reported to Child and Family Services authorities as required by Manitoba law. Similarly, if there is imminent risk of harm to oneself or others this will be reported to the appropriate authorities.

16. Dissemination/Withdrawing - Feedback

Member Checking

Will you be providing participants with the opportunity to review their data?	No

Dissemination

How will information from or about your participants be presented (e.g., summary statistics for the whole group, direct quotations from their interviews)?	Direct quotations from the interviews, quotations from focus group and partner meetings discussions, summary statistics for the whole group, thematic analysis of qualitative data.
In oral and/or written dissemination, will you refer to individual participants?	Yes
How will you do so (e.g., by their real name, by a pseudonym, by a general descriptor)? If using pseudonyms/general descriptors, indicate the naming convention that will be used.	Participants' names will be replaced by a pseudonym or number in representing their quotes from qualitative interviews. Quotations from focus group dialogue may also be published or presented using general descriptors (e.g., “female participant”).
What is your studys dissemination plan? Include who you will engage with and how you will engage with them (e.g., publications, presentations, articles, reports, theses, website, videos, creative works, community meetings).	The findings from this research study may be disseminated through various channels, including academic publications such as journal articles and conference presentations, as well as in thesis-related work. Additionally, the results may be shared in public forums, such as colloquia, to reach a broader audience. The intended purpose of disseminating these results is to contribute to the academic discourse, inform relevant stakeholders, and potentially influence policy and practice in the field.
Provide your plans for sharing the results of the research with your participants. Participants should be given a choice in how they wish to receive this information and approximately when to expect it (MMYY).	The feedback will be provided by the research team and delivered to participants in a format of their choice, such as email or postal mail. The research team will ensure that the summary is accessible and understandable to participants. The summary is expected to be provided approximately by: # Adults 55+ Focus Group - December, 2025. # Community Providers Focus Group - March, 2026. # Adults 55+ CONNECT - March, 2028. # Community Providers IKT - March, 2028.

17. Dissemination/Withdrawing - Withdrawing

How and when will you inform participants about the withdrawal process?	Participants are informed about their freedom to withdraw prior to the commencement of data collection, during the signing of the consent form.
Indicate what participants should do to withdraw from the study at any point. Include information on who they contact and how the contact should be made.	If a participant chooses to withdraw, they must inform the study coordinator or lead investigator by contacting them within one month of their participation. Training and Facilitation component. If a facilitator decides to withdraw before completing a program cycle, they are asked to notify the Research Coordinator at least two weeks in advance, to allow time for alternate arrangements and to ensure that compensation for completed sessions can be processed accordingly.
Indicate what will be done with the participants data when they withdraw.	Beyond one month of their participation, data may have been analyzed and summarized, and withdrawal will no longer be possible.
Is there a deadline after which the nature of your data analysis would make it impossible for participants to withdraw? Please provide a MMYY.	Within one month of the participation in each participant group.

18. Dissemination/Withdrawing - Other Approvals

Who are the group(s)/organization(s) you will require approval from and how will you obtain this approval?

In the study, four community sites in Manitoba, Saskatchewan, New Brunswick, and British Columbia, A & O: Support Services for Older Adults, have committed to offering The CONNECT Program to adults ages 55+ through telephone-based format as well as virtually (e.g., Zoom video) to meet organizational mandates and interests. We have received letters of support from these organizations, confirming their willingness to participate in this research. For collaboration in our research, organizations have confirmed that obtaining research ethics approval from their institutions is not required.

Attach any scripts that will be used to request permissions/approvals. Copies of communications or approval letters may also be attached. All attachments should be uploaded as individual documents.

Type	Name	Document
Other Approval Documents	Letters of Support from Partner Organizations	Letters of Support from partner organizations.pdf
Other Approval Documents	Adults 55+ CONNECT Letter of information to partners	Adults 55+ CONNECT Letter of information to partners.docx
