# Supplementary material for: Evaluating and implementing The CONNECT Program—A group-based telehealth intervention to reduce social isolation, loneliness, and mental health symptoms in adults 55+ vs routine community programming: Study protocol for a randomized controlled trial
Source: PLoS One. 2025 Nov 11;20(11):e0336031. doi: 10.1371/journal.pone.0336031 (PMC12604767; doi:10.1371/journal.pone.0336031)
Supplement: S4 File — (DOCX) [file pone.0336031.s004.docx]

# **Metadata**

Table 1. The structured summary of trial design and methods follows the items outlined in the World Health Organization Trial Registration Data Set (version 1.3.1) [1]

| **Primary registry and trial identifying number** | **ClinicalTrials.gov, NCT07107906** |
| --- | --- |
| **Date of registration in primary registry** | August 2025 |
| **Source of Monetary or Material Support** | Canadian Institutes of Health Research |
| **Primary Sponsor** | University of Manitoba |
| **Contact for public queries** | Kristin A. Reynolds, Kristin.Reynolds@umanitoba.ca |
| **Contact for scientific queries** | Kristin A. Reynolds, University of Manitoba, Winnipeg, Manitoba, Canada |
| **Public title** | The CONNECT Program: Engaging Community Organizations in the Implementation and Evaluation of a Group Telehealth Mental Health Program for Older Canadians |
| **Scientific title** | Evaluating and Implementing The CONNECT Program — a Group-Based Telehealth Intervention to Reduce Social Isolation, Loneliness, and Mental Health Symptoms in Adults Ages 55+, Compared to Routine Community-Based Programming: Study protocol for a Randomized Controlled Trial |
| **Countries of recruitment** | Canada |
| **Health condition(s) or problem(s) studied** | Social Isolation, Loneliness, Anxiety, Depression |
| **Interventions** | Intervention: The CONNECT program |
|  | Comparator: routine community-based programming |
| **Key inclusion and exclusion criteria** | Age of eligibility for study: ≥ 55 years old  Inclusion Criteria: able to speak, read, and understand English; can manage hearing or vision challenges well enough to participate in group conversations; self-reported experiences of loneliness, social isolation, and/or mental health challenges (e.g., anxiety or depressive symptoms)  Exclusion Criterion: cannot communicate in English (verbal or written) |
| **Study type** | Intervention |
|  | Allocation: Randomized  Intervention Model: Crossover  Masking: Outcome Assessor, Data Analyst |
|  | Primary purpose: Treatment |
|  | Effectiveness and Implementation |
| **Date of first enrolment** | September 2025 |
| **Target Sample Size** | 128 |
| **Recruitment status** | Recruiting |
| **Primary Outcomes** | Psychological Flexibility |
| **Key Secondary Outcomes** | Loneliness, social isolation, emotional support, symptoms of anxiety, symptoms of depression, and mental health literacy |
| **Other pre-specified outcomes** | Acceptability, Adoption, Appropriateness, Feasibility, Implementation Costs, Penetration, Sustainability of The CONNECT Program |
| **Ethics Review** | University of Manitoba, Research Ethics Board 1  Status: Approved  Date of approval: 07/22/2024 |
| **IPD sharing statement** | IPD will not be shared. Only aggregate results will be disseminated through publications and presentations. |

**Protocol Version**

August 2025, Version 1

**Names, affiliations, and roles of protocol contributors**

Kristin A. Reynolds, Department of Psychology, University of Manitoba, Winnipeg, Manitoba, Canada – Principal Investigator

Kira Kudar, Department of Psychology, University of Manitoba, Winnipeg, Manitoba, Canada – Research Coordinator

Georgia Gopinath, Department of Psychology, University of Manitoba, Winnipeg, Manitoba, Canada – Research Assistant

Alex Ross, Department of Psychology, University of Manitoba, Winnipeg, Manitoba, Canada – Research Assistant

Inga Christianson, Department of Psychology, University of Manitoba, Winnipeg, Manitoba, Canada – Research Assistant

Alina Sanina, Department of Psychology, University of Manitoba, Winnipeg, Manitoba, Canada – Research Assistant

Robert Balshaw, Department of Community Health Sciences, University of Manitoba, Winnipeg, Manitoba, Canada – Co-Investigator

Lesley Koven, Department of Clinical Health Psychology, University of Manitoba, Winnipeg, Manitoba, Canada - Co-Investigator

Corey S. Mackenzie, Department of Psychology, University of Manitoba, Winnipeg, Manitoba, Canada – Co-Investigator

Nancy Newall, Department of Psychology, Brandon University, Winnipeg, Manitoba, Canada – Co-Investigator

Kathryn Sibley, Department of Community Health Sciences, University of Manitoba, Winnipeg, Manitoba, Canada – Co-Investigator

Stacey Miller, A & O: Support Services for Older Adults, Winnipeg, Manitoba, Canada – Community Partner, Knowledge User

Edwin Chau, Brella Community Services Society, Surrey, British Columbia, Canada – Community Partner, Knowledge User

Ronda Wedhorn, Senior Citizens Assistance Program, Moose Jaw, Saskatchewan, Canada – Community Partner, Knowledge User

Lyne Ouellet, Department of Gerontology, St. Thomas University, Fredericton, New Brunswick – Community Partner, Knowledge User

**Name and contact information for the trial sponsor**

Kristin A. Reynolds, Department of Psychology, University of Manitoba, 190 Dysart Road

University of Manitoba, Winnipeg, Manitoba, Canada, R3T 2N2. Email: Kristin.Reynolds@umanitoba.ca

**Role of Trial Sponsor**

The funder has no role in design, conduct, analysis, or reporting.

**Roles and responsibilities**

Study Principal Investigator, the Co-Investigators, the Research Coordinator and Research Assistants are responsible for the trial planning and design, trial execution, study protocol development and revision, ethics application submissions, participant recruitment, administration of The CONNECT Program, adverse events monitoring and reporting, trial master file management, budget oversight, data verification, statistical analysis and interpretation, publication of study findings.

Community Partners and Knowledge Users involvement is critical to the co-design of the study, recruitment of participants, delivery and facilitation of The CONNECT Program, and interpretation of results in the context of community-based service delivery.

No formal Data Monitoring Committee (DMC) due to low risk. PI and research team will provide ongoing oversight and daily activities. Data managed at University of Manitoba.

***Open Science***

**Trial Registration**

The trial was registered on ClinicalTrials.gov: NCT07107906, 08/05/2025, <https://clinicaltrials.gov/study/NCT07107906>.

**Access to Protocol and Analysis Plan**

The full trial protocol is available on ClinicalTrials.gov <https://clinicaltrials.gov/study/NCT07107906>. The statistical analysis plan will be published as part of this manuscript.

**Dissemination Plan**

Results will be disseminated via academic publications, conferences, and public presentations to inform research, practice, and policy.

**Reference**

[1] World Health Organization. WHO Trial Registration Data Set (Version 1.3.1). https://www.who.int/tools/clinical-trials-registry-platform/network/who-data-set
